# Health and Disease

**Published:** 1869-10

**Authors:** 


// HALL'S

JOURNAL OF HEALTH.

VOL. XVI. OCTOBER, 1869. No. 10.

### HEALTH AND DISEASE.

As ceaseless as the flow of time, do multitudes of rivers empty themselves into the lakes of the North, and passing over the Falls of Niagara, move onward to the boundless sea; but if the outlet of the St. Lawrence were closed, the accumulated waters would soon pass their limits, and devastate the Union, from the shores of the Atlantic to the mountains of the West. While steam is generating in a boiler, and as regularly passes off to expend its force on the moving machinery, all is well, and if arrested in its progress, may be harmless for a moment, but the next moment witnesses a fearful havoc. Coming down to things more familiar, to illustrations which meet every capacity, if water be constantly poured into a common vessel, and is allowed an equal exit, no harm follows; but if all egress be prevented, there must be a "running over," and if that is not allowed, and the stream is forced in, the receptacle will be shivered to atoms.

It would seem that a point so plain as this needs no illustration, and that wherever the principle could be applied in practical, everyday life, it would not fail to be attended to by any considerable class of persons, of even ordinary intelligence.

It is from the habitual failure to act out this almost intuitive truth, that three fourths of all the diseases arise which torture the body, enfeeble the mind, and waste the life of civilized man.

Three fourths of all our ailments occur, or are kept in continuance, by preventing the daily food which is eaten, from passing out of the body, after its substance has been extracted by the living machinery, for the purpose of renovation and growth. A healthy laboring man will eat daily two pounds of solid food, of meal, bread, vegetables and fruit; these two pounds, if brought together in one heap, would fill to overflowing the largest sized dinner-plate, and yet there are myriads of grown-up men and women to whom the idea has never occurred, that if this mass is retained in the body, day by day, inevitable harm must accrue.

The question, What becomes of it? seems never to have occurred, or to have been definitely or intelligently answered. If a man eats two pounds daily, nearly two pounds daily must in some way or other pass from his body, or disease and premature death is a speedy and inevitable result.

Taking food into the body is called eating, passing it from the body is called defecation, but for this term the phrase, "action of the bowels," will be used in these pages, and the expression, "daily action of the bowels," will mean a resort to the privy, back-house, etc., in every twenty-four hours. The ancients termed it, "visiting the Temple of Cloacina." An eccentric Western divine, in his rehearsals of what he had seen in railroad cars on his first visit to the East, designated them to his wondering parishioners in our hearing, as "distress houses." By whatever name called, the idea is distinctly presented to the reader's mind.

If the body is in good health, and the instincts of Nature are not suppressed, there is a proper proportion between the amount received and the amount passed out from the system. The very moment that such proportion is altered, disease begins in all constitutions, and all circumstances; nay, every additional hour of its continuance, that disease becomes more aggravated, more destructive, more difficult of arrest, and more certain of disaster and of death. The object of passing food through the body is threefold in youth; in maturity, two; for growth, sustenance, and repair in the one, in the latter for support and repair only, that is, nutrition; and the process by which the system separates nutriment from the food is called digestion; the distribution of this digested material to the different parts of the body where needed, for the purpose of being incorporated into bone, flesh, nerve, and tendon, is termed assimilation.

The power which set the universe in motion, ordained that it should be kept in motion by an inherent property; this we call "gravitation." That same power started the complex machinery of corporeal man, and endowed it with a capacity of continuance to the full term of animal life; this we call "instinct."

The irresponsible brute has no other guide to health, than that of instinct; it is in a measure absolutely despotic, and can not be readily contravened.

By blindly and implicity following this instinct, the birds of the air, the fish in the sea, and four-footed beasts and creeping things live in health, propagate their kind, and die in old age, unless they perish by accident or by the warfares which they wage against one another; living, too, from age to age without any deterioration of condition or constitution; for the whale of the sea, the lion of the desert, the fawn of the prairie, are what they were a thousand years ago; and that they have not populated the globe is because they prey on one another, and man in every age has lifted against them an exterminating arm. Man has instinct in common with the lower races of animal existence, to enable him to live in health, to resist disease; but he has in addition a higher and a nobler guide—it is Reason. Why he should have been endowed with this additional safeguard, is found in the fact, that the brute creation are to be used for temporary purposes, and at death their light goes out forever, but man is designed for an immortal existence, of which the present life is the mere threshold. He is destined to occupy a higher sphere, and a higher still, until, in the progress of ages, he passes by angelic nature; rising yet, archangels fall before him; and leaving these beneath and behind him, the regenerated soul stands in the presence of the Deity, and basks forever in the sunshine of his glory.

Considering then, that such is his ultimate destination, it is no wonder that, in his wise benevolence, the great Maker of us all should have vouchsafed to the creature man, the double safeguard of instinct, and of a diviner reason; that by the aid and application of both, his life might be protracted, and protected too, under circumstances of the highest advantage and most extended continuance, in order to afford him the fullest opportunity of preparing himself for a destiny so exalted, and for a duration of ceaseless ages.

"A dying man can do nothing easy," were the last words of the immortal Franklin. A diseased man can do nothing well, are words of our own, quite as true.

If any thing should be well done, it should be the preparation which is needed to fit us for the exalted condition which has just been described, and to do it well, the highest health, and the longest life should be sought by all. Such a preparation should be made under the most favorable of all possible conditions, and it is to no less an end, that this book has been conceived, to wit, to show the reader how health may be maintained, and how disease may be averted to the utmost limit of human life, that by the aid of health and length of days, the most perfect preparation possible may be made for the immortal existence beyond, and in this light, who shall deny that Health is a duty? Echo answers, "Disease is a crime!"

A few men in every age have been found, who by obeying instinct in the light of reason, have lived in health to the age of seventy, eighty, and a hundred years. Silliman, and Nott, and Humboldt, and Hamel, died among their nineties, living examples to us of what has been done. And what has been done by a few, may be done again by uncounted millions of our kind, if the work of temperance be commenced in the prime of youth. Young man, or maiden, under twenty, we implore you to begin that temperance this hour, in the light of these pages. But mind you, we do not mean a temperance restricted in its application to spirituous drink, but on the comprehensive scale laid down in the Holy Scriptures, in the injunction to be "Temperate in all things." While it is quite certain that those who begin in their teens to adhere to a rational temperance, may very safely calculate on reaching three-score years and ten, and even four-score, there is the hope which example and uncontroverted fact give, that even if health be lost at "forty-five," a wise temperance begun and continued from that age, promises the living in comfort and happiness, to double the number of years!

Lewis Cornaro, an Italian nobleman, gifted and rich, yielded to the depravities of his nature, and at the early age of forty-five, found himself a wreck in fortune, fame, and health. The physicians whom he consulted, being familiar with his excesses and his reckless character, fortified in their opinion by the evident fearful inroads which disease had made on his constitution, considered an attempt at restoration so hopeless, that they declined bending their minds to the preparation of a proper prescription; and to save themselves, as they supposed, a useless trouble, they informed him that he was beyond remedial means, and that the best thing he could do would be to reconcile his mind to the inevitable event, and make for it a Christian preparation.

He at once determined that, as he had but a short season to live, it should be a merry one, and was about casting himself into the maelstrom of a drunken vicious life, but by some unexplained circumstance, a freak possessed him, that at one effort he would cheat death and the doctors, by entering at once upon a life of the most heroic self-denial, and become in all respects a temperate man. So precise was he, that he weighed his food, and measured his drink, to the end of his life. He regained his health, regained his possessions, resumed his title, and his social position, and became a happy-hearted, Christian-minded gentleman. His whole nature seemed to overflow with kindness to all his race. On the twelfth of March, fifteen hundred and sixty-five, feeling that he was approaching the termination of life, while reclining on his cot, the excellent old man exclaimed: "Full of joy and hope I resign myself to thee, most merciful God." He then disposed himself with serenity, and closing his eyes as if about to slumber, gave a gentle sigh, and expired at the age of "ninety-eight years."

On a sunny October morning in eighteen hundred and forty-four, while in Edinburgh, we took breakfast at his own house with George Combe, then in the height of his reputation, as the champion for the promotion of the general health of the people. Six years earlier we had met him in New-Haven; during that interval, he seemed to have lost nothing in vigor of body or of mind. At his own table, his conversation ran freely on his favorite topics, how to improve the general health, and thereby add largely to the happiness of the whole human family. At the time we speak of, his mind was occupied with two subjects, aiding his shoe-maker to construct a sole which should best support the arch of the foot, and add to that natural spring which is so essential to sprightly locomotion; and the construction of houses in a manner best calculated to secure a thorough ventilation. Andrew Combe was in the country at the time, but was having a house built for himself according to the views of his brother George, who took us through this building, and with kindly interest pointed out the contrivances which he had fallen on to keep the house perfectly dry, and as nearly as possible to secure a constant supply of fresh air from without, for the use of its intended occupants. Here was one of the finest minds, and one of the kindest hearts in Scotland, in England, in the world, busying itself for the amelioration of the condition of mankind in various publications; doing it too for purposes of pure benevolence—that very benevolence which had already made him a fortune in the practice of law, not in the fashion of the present hour, when needy attorneys, with utter recklessness of all honor, urge on confiding clients to contests in the court-room which they know will be disastrous, for the sake of a paltry fee to fill a lank purse and a famishing stomach; not in the low and cowardly abuse of an honest citizen, for the sake of averting condign punishment from some heartless gambler or sneaking thief, or viler slanderer, the presence of the judge affording a rampart behind which he may cower, and save himself; nor did he secure his ample estate in fleecing the widow and the orphan child, by taking advantage of their unprotected condition, of their ignorance of their rights, and of their confiding nature, to transfer to a remorseless maw the lion's share of the means which a dead husband and father's industry, forethought, and economy, had provided for the helpless ones whom he had so dearly loved in life. No! no! by none of these base means, did George Combe retire on an ample competence. He did it in the legitimate practice of a profession, whose honor and whose glory it is, to protect the unprotected, to save the innocent, and to defend right against might; he did it by a conscientious and just management of the estates of widows and children, and in endeavoring to bring his neighbors and fellow-citizens to a settlement of their controversies by friendly arbitration.

But what led the profound scholar and the rich lawyer to busy himself in devising means for the improvement of the general health of the people? It was from having had his own constitution, in common with other members of his father's family, so impaired in early life, as to make him familiar with bodily disease and pain and suffering. He had been ignorantly brought up in a locality, and in habits of life, which early bent his manly form, and turned his hair to snowy whiteness, before he reached his fiftieth year. By living in a vitiated air, by the habitual neglect of ventilating clothes, beds and bedding, of bodily ablutions, when "warm water was constantly at hand," his constitution was so impaired in early life, that his health ever after remained infirm, and yet by a wise attention to the laws of life, his frail system was sustained, and he survived in considerable health, in active, efficient usefulness to a good old age, dying within a month of this present writing; having almost reached his eightieth year.

His brain remained healthy and clear, his mind unclouded to the last day of life, his manuscript within a fortnight of his death, being "in the clear firm hand of twenty years ago." This, upon reflection, will be found a more striking illustration than *Cornaro's*, of the value of wise habits of life, without any medicine whatever, in arresting the progress of disease, and continuing existence under circumstances of considerable comfort, to a good old age. For Cornaro had a constitution so vigorous, that for twenty years it held out against a life of profligacy and debauchery; whereas in the case of Dr. Combe, his constitution was prematurely impaired, at an age when the Italian's was in its undiminished prime.

We have known useful, honest, and Christian men plunge themselves into irretrievable ruin, lose their all, and die broken-hearted, leaving whole households in immeasurable affliction, from having been induced by vagabond lawyers to swear to phrases of expression which were not literally true, under the pretense and assurance that it was merely a legal form, and meant nothing, but which through the searching scrutiny of vindictive prosecution, could not be classed otherwise than as constructive perjury. One of the most useful members of society as now constituted, is a conscientious, honorable, and profound lawyer; but to have to do with those not so, has brought ruin to the purse, to the peace, to the health and life of multitudes; and if by this digression we have put some of our readers on their guard against going to law prematurely, or for slight causes, or, if compelled into it by the rascality of unprincipled men, to take no legal advice except from those "known of all" to be high-minded members of their profession; and not even then to subscribe to any assertion which is not to their own unsophisticated comprehension, a literal truth with a large margin. If we say, we do this, we shall have accomplished a humane work, and to some extent have aided in banishing from our social circles, and from official associations with honorable men, a brood of blood-thirsty har pies, who are a living disgrace to their kind. Therefore, as a means of health, avoid the court-house, endure reproach, arbitrate, suffer wrong, do any thing not dishonorable, sooner than enter into the hateful precincts.

Two things are necessary to health and long life, under all conceivable circumstances—the proper regulation of the diet, and the proper regulation of the bowels. As to the former, there is no danger that the wants of the appetite will be neglected. All eat full enough for the needs of the body. We therefore turn our attention to the proper regulation of the bowels, as having a greater bearing on human health and disease than almost all other things together, in that it requires a daily attention by "all of women born," an attention as imperative in disease as it is in health, for in all human maladies it is the first care of the physicians. When the bowels do not act with sufficient freedom, when less is passed from the body than is received into it, it is termed costiveness, but when there are no discharges for days together, that is called constipation. It will be readily seen that one runs into the other, and the distinction is not important; for the great idea is, that whenever twenty-four hours have passed, during which the bowels have not acted, disease has begun; there is a cause for it, and the truly wise will take measures for the removal of that cause, and for remedying the effect. In passing, we state a general rule of safe application.

When a person is in the habit, regularly, of having an action of the bowels about a certain hour every day, and that hour passes without such action, eat not an atom of any thing until such action is had, but drink as much pure water, or warm teas of any kind as there may be inclination for, and keep moving freely in the open air for the greater part of the time. If at the end of forty-eight hours the bowels are still bound, consult a sober, thoughtful, conscientious physician, and swallow not even the simplest substance in nature as a medicine, without his advice. We do not say without his permission, but throw the whole responsibility upon him, and let him originate the prescription.

The young, if let alone, are as regular as a clock, almost, in their daily evacuations, but comparatively few, especially in cities, and in large towns, reach the age of twenty years, without having this regularity interfered with; and what we want above all other objects in these pages is, to point out to the attention of the young, the manner in which constipation first begins to gain a footing in the system, preparatory to a permanent hold, to the undermining of the health, and of making a blast and a blight of human hopes and happiness. The lower portion of the bowels is called the *Rectum*, "right," or "upright," from its erect position in the body; it is some half a dozen inches long; it is one, two, several inches in circumference, according to age, sex, and habits of life. The point at which it opens out of the body is called the *Anus*, which opens and closes as occasion may require—very much like a miller's bag, which operation it performs first by an instinct which produces a "desire," which is telegraphed to the brain through the nervous "wires" of the body; at this point, the will comes to the aid, the function is performed; the very pleasure of that performance, by the ease which it gives, is not the least among the countless beneficences of our Creator, in that he has arranged a certain degree of gratification to attend those operations of nature which are accounted the most ignoble.

But suppose the intimation is disregarded, then it is made more decisive every moment, until actual pain gives a note of warning not likely to be neglected. If, however, these indications are not attended to, the delicate sensibilities of the parts are blunted, their keen edge dulled, and mischief follows apace.

The young are modest and impatient. They are often led to disregard a call of nature, to resist an inclination to stool; something makes it inconvenient at the time; a friend is present who expects to depart in a few moments, but the departure is protracted; a lesson is to be studied, a chapter is to be completed; a few pages will end a book, an unexpected sight is presented, a strange occurrence takes place, it is raining, a newspaper article is to be completed, a story is to be heard out, a job is to be finished, or, being from home, no convenient place is at hand; in these and many other circumstances, the feeling is, that "in a minute or two" the obstacle, real or imaginary, will be removed, and nature will be attended to; so by an effort, the inclination is repressed, the mind lays hold on something else, its attention is compelled away, and desire is for the present gone. But nature has been violated, and, as in other cases, when what ought to be accomplished in one way can not be done, she directs her attention to the next most practicable plan for bringing about her purposes, as follows. The "Rectum" is a distensible purse, or pouch, or bag. A meal-bag may be filled with bran, but by careful packing may be made to hold a great deal more, because it is of a flexible material. The rectal bag is so contrived, that when it is "full enough," it calls for being discharged, as a ship does, when it has safely reached its destined haven. Its sensibilities are so nicely adjusted, that the very moment enough material has been passed into it, from the upper portion of the body, to cause a certain amount of distention, it contracts upon itself, just as a partly-filled bladder is discharged, if compressed by the fingers of the hand; this is a wonderful contrivance, and the principle is brought to bear with admirable advantage in some of the complicated machinery of modern times, in portions of them where a piston, or arm, or wheel, advances to a certain point, and then recedes in the opposite direction, by no possibility going the tenth part of an inch beyond its appointed boundary: but, transcending any mere machine of human contrivance, which moves by an inevitable law, and can not possibly have a variation, the organisms of the human body have an adaptability to emergencies, approaching to a discretion, which shows that their great Artificer is God. So the rectum is made distensible, as there are cases where its immediate discharge may be impracticable, or the health of the system requires a delay. In cholera, for example, if the discharges are prevented, or retarded under proper conditions, the patient is saved. If there be a valid reason under any circumstances which measurably justifies temporary resistance to a call of nature, the rectal bag is capable of adapting itself to the occasion, and can receive a large surplus beyond what it was intended to hold ordinarily. But a person postponing a call should, the instant the circumstances have passed which seemed to require the postponement, repair to the privy, wait, and solicit the return of the call—not wait for it to return itself, for that call may not be repeated for hours, or even days. Nature does not suffer violence with impunity.

There is almost as great a tendency to mathematic regulari ty in the workshop of the body, as there is in the revolution of the stars. Therefore, if a call of nature is resisted for an hour to-day, it will most likely be an hour later than usual tomorrow; but then it does not come so strong, it is less and less full day after day, until the whole machinery is interrupted, proportions are changed, and that is disease.

It is a hazardous act to resist the call of nature for any cause, for a single moment, and the hazard should be removed as just indicated, by immediate attention to the method named—invite the return.

